# Application of the WHO new vaccine introduction prioritisation and sequencing framework to guide evidence-based vaccine introduction decisions in Iran, 2025–2030

**DOI:** 10.1136/bmjopen-2025-115580

**Published:** 2026-04-15

**Authors:** Akbar Fotouhi, Sobhan Younesian, Seyede Maryam Mousavi, Seyed Mohsen Zahraei, Sussan Mahmoudi, Farid Fotouhi, Marzieh Nojomi, Maryam Alavi, Omid Zamani, Iraj Sedighi, Alireza Nateghian, Ali Akbari Sari, Masoud Movahedi, Mohamad Gharagozlou, Setareh Mamishi, Mohammadreza Salehi, Alireza Biglari, Ali Es-Haghi, Ghobad Moradi, Mohammad Mehdi Gouya

**Affiliations:** 1 Department of Epidemiology and Biostatistics, School of Public Health, Tehran University of Medical Sciences, Tehran, Iran (the Islamic Republic of); 2 School of Medicine, Tehran University of Medical Sciences, Tehran, Iran (the Islamic Republic of); 3 Department of Vaccine Preventable Diseases, Center for Communicable Diseases Control, Iran Ministry of Health and Medical Education, Tehran, Iran (the Islamic Republic of); 4 ENT and Head and Neck Research Center and Department, The Five Senses Health Institute, School of Medicine, Iran University of Medical Sciences, Tehran, Iran (the Islamic Republic of); 5 Preventive Medicine and Public Health Research Center, Psychosocial Health Research Institute, Department of Community and Family Medicine, School of Medicine, Iran University of Medical Sciences, Tehran, Iran (the Islamic Republic of); 6 Department of Communicable Diseases, World Health Organization Country Office for Iran, Tehran, Iran (the Islamic Republic of); 7 Department of Pediatrics, Faculty of Medicine, Hamadan University of Medical Sciences, Hamadan, Iran (the Islamic Republic of); 8 Department of Pediatrics, Iran University of Medical Sciences, Tehran, Iran (the Islamic Republic of); 9 Department of Health Management and Economics, School of Public Health, Tehran University of Medical Sciences, Tehran, Iran (the Islamic Republic of); 10 Department of Allergy and Clinical Immunology, Children’s Medical Center, Tehran University of Medical Sciences, Tehran, Iran (the Islamic Republic of); 11 Pediatric Infectious Disease Research Center, Tehran University of Medical Sciences, Tehran, Iran (the Islamic Republic of); 12 Department of Infectious Disease, Children’s Medical Center, Tehran University of Medical Sciences, Tehran, Iran (the Islamic Republic of); 13 Research Center for Antibiotic Stewardship and Antimicrobial Resistance, Department of infectious diseases, Imam Khomeini Hospital Complex, Tehran University of Medical Sciences, Tehran, Iran (the Islamic Republic of); 14 Department of Medical Genetics, Children’s Medical Center, Tehran University of Medical Sciences, Tehran, Iran (the Islamic Republic of); 15 Department of Physico Chemistry, Agricultural Research, Education and Extension Organization, Razi Vaccine and Serum Research Institute, Karaj, Iran (the Islamic Republic of); 16 Health Metrics and Evaluation Research Center, Research Institute for Health Development, Kurdistan University of Medical Sciences, Sanandaj, Iran (the Islamic Republic of); 17 Center for Communicable Disease Control, Iran Ministry of Health and Medical Education, Tehran, Iran (the Islamic Republic of); 18 Department of Infectious Diseases, Iran University of Medical Sciences, Tehran, Iran (the Islamic Republic of)

**Keywords:** PUBLIC HEALTH, Preventive Health Services, Vaccination

## Abstract

**Abstract:**

**Objectives:**

To document the first application of the WHO New Vaccine Introduction Prioritization and Sequencing Toolkit (NVI-PST) in the WHO Eastern Mediterranean Region and to describe how Iran’s National Immunization Technical Advisory Group (NITAG) adapted and implemented the framework to develop a prioritised roadmap for vaccine introduction during 2025–2030.

**Design:**

Policy implementation case study applying a structured multicriteria decision analysis-informed prioritisation framework through a three-phase process including framework adaptation, evidence synthesis, ordinal ranking of candidate vaccines, weighted aggregation and development of sequencing scenarios.

**Setting:**

National immunisation governance process in Iran, coordinated by the Ministry of Health and Medical Education and Iran’s NITAG, with technical support from the WHO Country Office.

**Participants:**

Core and non-core members of Iran’s NITAG and key immunisation stakeholders involved in the deliberative prioritisation process.

**Results:**

Human papillomavirus (HPV) vaccine ranked highest in both importance and feasibility, followed by pneumococcal conjugate vaccine (PCV) for high-risk adults and seasonal influenza vaccine for high-risk groups. Two sequencing scenarios were proposed: both placed HPV first, with either PCV or influenza third after the already-approved hexavalent vaccine. Respiratory syncytial virus (RSV) and varicella vaccines were classified as low priority for the 5-year horizon. The toolkit enabled structured multistakeholder deliberation, improved the transparency and reproducibility of prioritisation, and supported systematic integration of epidemiological, economic and programme evidence. The main implementation challenges arose from national evidence constraints, particularly gaps in adult RSV and pneumococcal disease burden, limited locally generated cost-effectiveness analyses and uncertainty in long-term budget impact estimation under macroeconomic instability, rather than from limitations of the toolkit itself.

**Conclusion:**

The NVI-PST proved feasible under national leadership and generated credible, consensus-based recommendations aligned with Iran’s public health priorities and programme constraints. Minor refinements (streamlined evidence compendium, simpler weighting, stronger secretariat support) would make the toolkit lighter and more sustainable, especially for resource-constrained settings. This Iranian experience provides a replicable model for structured multi-vaccine prioritisation in the Eastern Mediterranean Region and beyond.

**Strengths and limitations of this study**

- This study applied a standardised, WHO-developed prioritisation and sequencing framework (New Vaccine Introduction Prioritization and Sequencing Toolkit) within a nationally led decision-making process, enhancing methodological transparency, reproducibility and alignment with international best practice for vaccine prioritisation.
- The use of predefined criteria, explicit weighting, structured evidence synthesis and anonymous electronic voting reduced dominance bias and increased consistency in multistakeholder deliberations compared with informal consensus-based approaches.
- The framework explicitly separated importance (public health impact) and feasibility (programmatic and financial considerations), allowing prioritisation and sequencing decisions to reflect real-world implementation constraints rather than relying on a single composite score.
- Evidence synthesis relied largely on secondary and publicly available data; gaps in locally generated epidemiological, economic and adult disease burden data limited the precision of scoring for some vaccines.
- The process was resource-intensive and time-intensive, requiring a dedicated technical team and multiple workshops, which may limit scalability or frequent repetition in settings with limited National Immunization Technical Advisory Group secretariat capacity.

## Introduction

 Immunisation ranks among the most effective and cost-effective public health strategies, playing a vital role in preventing infectious diseases and decreasing morbidity and mortality.^1^ A recent article reports that, since its inception in 1974, the Expanded Programme on Immunization (EPI) has helped prevent more than 150 million deaths globally through widespread vaccination.^2^ Despite these benefits, countries encounter major obstacles when introducing new vaccines. The growing number of available and developing vaccines can overwhelm health systems, mainly because of limited resources. As a result, they must carefully prioritise which new vaccines to introduce and plan accordingly. In most countries, National Immunization Technical Advisory Groups (NITAGs) are responsible for providing evidence-based recommendations to guide vaccine introduction.^3^ In 2025, the WHO Strategic Advisory Group of Experts on Immunization (SAGE) urged NITAGs to look beyond individual vaccine recommendations and instead assess evidence across vaccines to prioritise those that best fit national health objectives.^4^

Developing evidence-based recommendations for vaccine prioritisation is a complex task. There are many possible criteria to consider, and determining which ones to use, as well as their relative weight, further complicates the process. Additionally, it is important to decide on which candidate vaccines should be compared. Several countries, including Bangladesh,^5^ China,^6^ the Republic of Korea^7^ and Thailand,^8^ have demonstrated that structured, multicriteria or consensus-based approaches can prioritise new vaccines. Yet these exercises vary widely in criteria sets, weighting schemes and decision rules, which limits transferability across settings and often yields a single composite rank that is less helpful for balancing feasibility with public-health importance or for sequencing introductions over time. These limitations underscore the value of a versatile, standardised yet adaptable approach to support transparent, context-sensitive prioritisation and sequencing.

Recently, the WHO, in collaboration with GAVI, UNICEF and the Bill & Melinda Gates Foundation, developed the New Vaccine Introduction Prioritization and Sequencing Toolkit (NVI-PST) to help countries make transparent, evidence-based decisions about which new vaccines to introduce and in what order.^9^ The NVI-PST provides a structured, multicriteria decision-making framework that guides NITAGs through a step-by-step process of (1) identifying candidate vaccines, (2) selecting and weighting assessment criteria, (3) collecting and synthesising relevant evidence and (4) ranking and sequencing vaccines based on importance and feasibility.

The toolkit complements existing WHO decision-support instruments, such as the Evidence-to-Recommendations (EtR) framework and the CAPACITI Decision-Support Tool, by integrating health-impact and programme feasibility considerations into a single decision process and by comparing candidate vaccines to select suitable ones for a country.

Early pilot applications of the NVI-PST have been reported in several countries, including Ethiopia, Bangladesh and China, where it enabled NITAGs to systematically compare multiple vaccine options and align recommendations with national priorities. However, no published report has yet documented its implementation in the Eastern Mediterranean Region. This gap provided a rationale for assessing its use in Iran.

Iran’s immunisation programme has long performed strongly, with near-universal EPI coverage and recent introductions of pneumococcal and rotavirus vaccines.^10^ Yet life-course vaccination is not fully embedded, creating gaps for adolescents and adults amid interest in options like human papillomavirus (HPV) and influenza. With finite resources, competing priorities and no common method for weighing importance against feasibility, decisions on what to introduce first are complex. The WHO NVI-PST offers a structured, transparent way to assess candidates and sequence introductions in line with national goals and programme capacity.

This study, therefore, applies the NVI-PST in the Iranian context to identify and prioritise potential new vaccines for introduction between 2025–2030, documenting both the benefits and the implementation challenges encountered during the process.

## Methods

### Study design and governance

**Figure 1:**
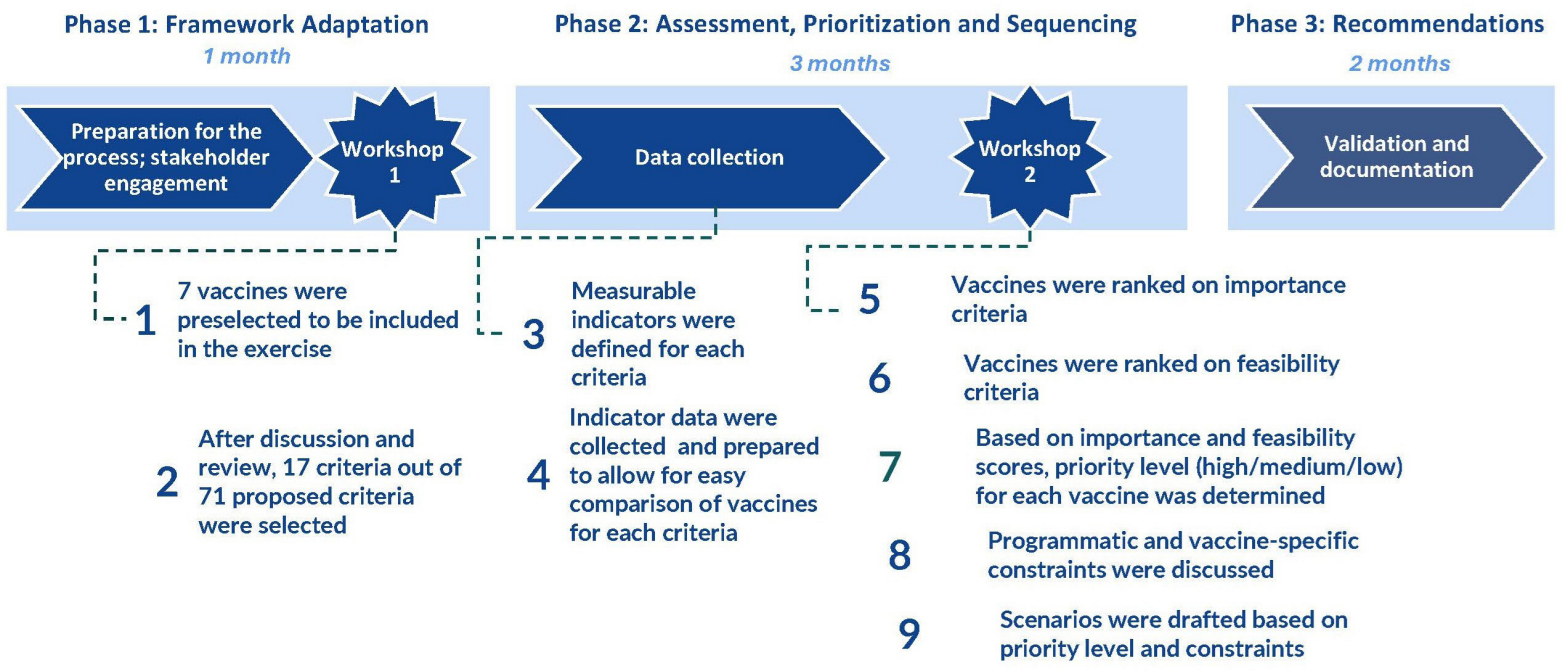
New vaccine introduction prioritisation and sequencing framework process. It has been adapted from NVI-PST. NVI-PST, New Vaccine Introduction Prioritization and Sequencing Toolkit.

This study is a policy implementation case study applying a structured multicriteria decision analysis (MCDA)-informed prioritisation framework within Iran’s national immunisation governance process. In practice, the WHO NVI-PST was implemented within Iran’s immunisation decision-making framework as a country-led initiative coordinated by Iran’s NITAG (I-NITAG) in collaboration with the EPI at the Ministry of Health and Medical Education (MoHME), with technical support from the WHO. A core implementation team, led by an I-NITAG member, was assigned to manage the planning, execution and documentation of the process. Team members facilitated workshops, developed voting tools, collected evidence and prepared supporting documents and presentations. The full implementation process is presented in figure 1.

### The NVI prioritisation and sequencing framework implementation

The NVI prioritisation and sequencing framework comprised three phases: (1) framework adaptation, (2) assessment, prioritisation and sequencing, and (3) development and dissemination of recommendations. I-NITAG was guided through a series of decisions to adapt the framework to Iran’s specific context and needs. This process included defining the time frame for the exercise, determining the list of vaccines to be considered, selecting criteria for comparing vaccines and establishing a weighting scheme for the selected criteria to inform decision-making (figure 1).

Initially, the toolkit proposed 21 preselected vaccines and 71 criteria for consideration. These criteria were identified through extensive systematic searches and reviews of existing tools, including the EtR and CAPACITI frameworks, with sources described elsewhere.^9^ The criteria encompassed three main dimensions: disease and vaccine characteristics, external factors and programme factors. They were subsequently ranked using three selection benchmarks: (1) the importance of the criterion, (2) data availability and (3) the criterion’s ability to differentiate among vaccines. This ranking yielded three categories: 16 essential criteria, 14 significant criteria and 41 other criteria. As described later, criteria with higher classifications were more likely to be included and were assigned greater weights. Additionally, the criteria were further classified into importance and feasibility criteria.

### Phase 1: framework adaptation

Phase 1 aimed to develop the workplan, engage stakeholders, define the exercise time frame, identify the list of vaccines for consideration and select decision-making criteria. Key decision-makers and stakeholders included core and non-core I-NITAG members, the EPI Director, the Head of the Center for Communicable Disease Control and in-country representatives from WHO and UNICEF.

Initially, stakeholders were introduced to the NVI-PST during a virtual session held on 25 February 2025. A total of 16 participants attended this session, including 11 core I-NITAG members, 5 non-core members and stakeholders. At the end of this meeting, participants were asked to vote on the exercise time frame, candidate vaccines and recommended criteria to be used in the process. Following this virtual meeting, another session was convened on 21 May 2025 as an in-person, 1 day meeting referred to as Workshop I, to finalise the framework implementation. A total of 21 participants attended Workshop I, including 10 core I-NITAG members, 11 non-core members and stakeholders. At the beginning of the workshop, the voting results were presented to participants to provide a basis for discussion. Participants then reviewed and deliberated on potential options and agreed on the following: (1) the time horizon for the new vaccine introduction, (2) seven preselected vaccines and (3) 17 prioritised criteria for consideration within the framework.

The toolkit recommended ranking the prioritisation criteria based on their importance, data availability, discriminative capacity and alignment with the national context, resulting in approximately 8–13 essential criteria, 5 significant criteria and 3 additional criteria, while maintaining a balance between importance and feasibility considerations. During Workshop I, the implementation team facilitated the discussions to ensure adherence to these guidelines. The resulting criteria were further subdivided into one or more indicators to obtain accurate and objective evidence for each criterion. These final indicators were subsequently presented to the stakeholders in another online meeting held on 8 June 2025 at the end of Phase 1.

### Phase 2: assessment, prioritisation and sequencing

#### Evidence collection and synthesis

The team lead coordinated the standardised evidence gathering and abstraction for each criterion-vaccine pair using published and grey literature, SAGE/WHO guidance and position papers, United States Centers for Disease Control and Prevention recommendations, WHO/UNICEF data systems, WHO Vaccine Evidence Compendium for assisting NITAGs, Global Burden of Disease estimates and programme records on epidemiology, service delivery capacity, financing constraints and market and pipeline intelligence for upcoming vaccines. For each pair, abstractors recorded study attributes (including authors, year, study design, setting and population), key outcomes and contextual notes. In cases of conflicting evidence, multiple sources were compared which provide a broader range of estimates. Evidence gaps and uncertainties were explicitly documented in the synthesis. Ultimately, the team lead summarised all the evidence and prepared it for presentation at Workshop II.

#### Prioritisation and sequencing

At the end of Phase 2, a second in-person meeting (Workshop II) was held over 2 days, on 19–20 August 2025. A total of 19 participants attended, including 11 core I-NITAG members and 8 non-core members and stakeholders. During the workshop, I-NITAG assessed the collected evidence, ranked vaccines for each criterion, discussed high-priority, medium-priority and low-priority vaccines, and developed two sequencing scenarios. On the 1st day, the evidence was presented to participants. For each criterion, the team lead summarised the evidence synthesis and facilitated discussions comparing vaccines, after which voting members independently ranked vaccine candidates for that criterion. The implementation team exported the results to a prespecified calculation model. For each criterion, participants assigned an ordinal rank to each vaccine candidate (1=highest priority). Rankings reflected relative positioning rather than absolute quantitative differences between vaccines. For each criterion, the mean rank across voting participants was calculated to obtain a collective ranking.

After criterion-specific mean ranks were derived, the rankings were aggregated across criteria to generate overall rankings. Two separate composite scores were calculated for each vaccine: one representing overall importance and one representing overall feasibility. Aggregation followed the weighting structure defined during Phase 1, whereby essential, significant and other criteria were assigned weights of 3, 2 and 1, respectively. These weights were derived from the NVI-PST framework classification of criteria based on their assessed importance, data availability and discriminative capacity. The weighting scheme was normatively defined during framework adaptation rather than empirically estimated.

Although ordinal rankings do not assume equal intervals between positions, mean rank aggregation is a commonly used approach in structured multicriteria deliberative processes to summarise collective preference patterns. The weighted mean rankings were used to position each vaccine on a two-dimensional matrix plotting importance against feasibility.

To assess the stability of results, the implementation team reviewed both weighted and unweighted rankings and examined clustering patterns within the importance–feasibility matrix during deliberation. While no formal quantitative sensitivity analysis was conducted, final prioritisation decisions were not based solely on marginal numerical differences but were informed by structured discussion and contextual judgement.

On the second day, the ranking results were presented, including a quadrant plot of weighted importance versus weighted feasibility to visualise clusters. The I-NITAG deliberated and classified vaccines as high-priority, medium-priority or low-priority, explicitly referencing the evidence, rankings and the Iranian programme context. To account for differing assumptions about product-market availability, programme capacity limitations and insurance coverage, two sequencing scenarios were developed for the prioritised vaccines. These scenarios constituted the final outputs of Phase 2 of the framework. At the conclusion of the workshop, I-NITAG established a schedule for reassessing the scenarios and repeating the prioritisation exercise, balancing analytical value with capacity constraints.

### Phase 3: development and dissemination of recommendations

Following Workshop II, the implementation team drafted a recommendations package, which included the two sequencing scenarios, the prioritised list of vaccines (high, medium and low), and evidence summaries by criterion and vaccine. Drafts were circulated to I-NITAG members for review and revision, discussed with the EPI and MoHME to ensure consideration in the National Immunisation Strategy, and subsequently finalised for submission to the MoHME for endorsement.

### Ethical considerations and quality assurance

This exercise relied on secondary data and stakeholder deliberation, without collecting individual-level data. The process emphasised transparency, thorough documentation of evidence, clear decision rationale and systematic archiving, in accordance with the I-NITAG Secretariat’s good-practice modules on documenting recommendations. Conflicts of interest were declared in accordance with I-NITAG procedures. Methodological adherence to the NVI-PST was ensured through the use of standardised slide decks, questionnaires and evidence templates; redundancy in evidence review by abstractors and the team lead; and the predefinition of decision rules and polling procedures. All voting activities were conducted via the Porsline platform (www.porsline.ir).

### Patient and public involvement

Patients and the public were not involved in the design, conduct, reporting or dissemination plans of this study, as such involvement was not applicable to the nature of the research.

## Results

### Decisions on time frame and vaccine preselection

From the initial 21 vaccines proposed by the NVI-PST, the implementation team found that five vaccines, including the hepatitis B at birth, *Haemophilus influenzae* type b, rotavirus, measles-rubella (as part of the MMR - measles, mumps and rubella - vaccine) and the diphtheria, tetanus and pertussis booster vaccines, were already included in Iran’s immunisation programme and were therefore excluded from further consideration. Subsequently, the implementation team added six additional vaccines to the preselection list. In total, 19 of the 23 WHO-recommended vaccines for all age groups were either already introduced in Iran (n=9) or included for evaluation in the current programme (n=10). Most of the remaining vaccines, such as those for Japanese encephalitis, yellow fever and tick-borne encephalitis, were primarily recommended for endemic regions (see table 1).

**Table 1:** WHO immunisation recommendations, NVI-PST vaccine suggestions and candidate vaccines for introduction

Recommended by WHO	Recommended by NVI-PST	Evaluated in the programme
BCG	–	Already introduced in Iran
Polio	–	
DTP-containing vaccine	DTP booster	
Hepatitis B	Hepatitis B	
*Haemophilus influenzae* b	*Haemophilus influenzae* b	
Measles	Measles—Rubella	
Rubella		
Mumps	–	
Rotavirus	Rotavirus	
HPV	HPV	HPV*
Malaria	Malaria	Malaria
Cholera	Cholera	Cholera
Typhoid	Typhoid	Typhoid
Dengue	Dengue	Dengue
–	Shigella	Shigella
–	Hexavalent	Hexavalent*†
–	Meningitis (multivalent)	Meningitis (multivalent)
–	Gonorrhoea	Gonorrhoea
–	RSV	RSV*
–	Ebola	Ebola
–	Chikungunya	Chikungunya
–	Mpox	Mpox
–	GBS	GBS
–	Tuberculosis (new vaccine)	Tuberculosis (new vaccine)
–	Hepatitis E	Hepatitis E
Hepatitis A	–	Hepatitis A
Pneumococcal (conjugate)	–	Pneumococcal for high-risk groups*
Meningococcal	–	Meningococcal group B
Seasonal influenza	–	Influenza for high-risk groups*
Chickenpox	–	Chickenpox*
–	–	Acellular pertussis*
–	–	Zoster (Shingles)
Rabies	–	–
Japanese encephalitis‡	–	–
Yellow fever‡	–	–
Tick-Borne encephalitis‡	–	–

* Preselected vaccines during Workshop I.

† It was included in the voting but later excluded because this vaccine was approved prior to the programme.

‡ Recommended only in endemic areas.

DTP, diphtheria, tetanus and pertussis; GBS, group B streptococcus; HPV, human papillomavirus; NVI-PST, New Vaccine Introduction Prioritization and Sequencing Toolkit; RSV, respiratory syncytial virus.

Following discussions during Workshop I, I-NITAG members established a 5-year time horizon for the programme and selected seven vaccines for evaluation: the HPV vaccine, the seasonal influenza vaccine for high-risk groups, the hexavalent vaccine, pneumococcal conjugate vaccine (PCV) for high-risk groups, acellular pertussis (aP) vaccine, respiratory syncytial virus (RSV) vaccine and the varicella (chickenpox) vaccine. The hexavalent vaccine had been approved by I-NITAG prior to the start of this project, but had not yet been implemented. During the course of the project, it began replacing the pentavalent vaccine. Consequently, although it was initially included and endorsed through the questionnaires, it was later excluded from the implementation framework due to its ongoing national rollout.

### Criteria selection

During Workshop I, the results of the preceding online meeting were presented to I-NITAG members and thoroughly discussed. By the conclusion of the workshop, participants re-ranked the criteria before finalising them. Following the voting process, four criteria were identified as overlapping with higher-ranked ones and were subsequently merged (1 criterion) or excluded (3 criteria) to eliminate redundancy and ensure comprehensiveness. Two additional criteria were excluded: one pertaining to the WHO prequalification status of the vaccines, which was uniform across all candidates and did not discriminate among them, and another that was considered too complex for adequate data collection. Among the criteria with tied rankings, certain importance criteria were omitted to achieve a more balanced distribution between importance and feasibility criteria. Ultimately, a final set of 12 importance and 5 feasibility criteria was selected, comprising 10 essential, 2 significant and 5 other criteria, which were further subdivided into 30 specific indicators (table 2 and online supplemental table 1).

**Table 2:** Top 29 ranked criteria and final selected criteria along with their category, weights and exclusion reasons

Rank	Category and weight*	Votes (%)	Criteria
**1**	**Essential**	**100**	**Vaccine effectiveness, including in different populations/age groups/cohorts**
**2**	**Essential**	**100**	**Mortality and lethality, including in different sociodemographic and age groups**
**3**	**Essential**	**100**	**Incidence in different sociodemographic and age groups**
**4**	**Essential**	**90**	**Risk at individual level, including type, severity, consequences and frequency of adverse event following immunization (AEFI), including reactogenicity profile and capacity to mitigate known adverse events**
**5**	**Significant**	**80**	**Disability-adjusted life years**
**6**	**Essential**	**80**	**Absence of satisfactory alternatives to prevent/treat the disease (considering effectiveness, cost and practicality)**
**7**	**Other**	**80**	**Long-term disease complications (eg, frequency of survivors with sequelae)**
**8**	**Essential**	**80**	**Market availability of the vaccine and supplies over the selected time period**
**9**	**Essential**	**70**	**Availability and sustainability of funding to cover the total cost of the programme (incl. GAVI eligibility)**
10	Significant	70	Efficacy and immunogenicity of the vaccine in the target population†
11	Essential	70	Prevalence across different sociodemographic and age groups‡
**12**	**Essential**	**50**	**Coverage of active serogroups or serotypes in the country (for serogroup-specific or serotype-specific vaccines)**
**13**	**Essential**	**50**	**Duration of protection and waning of immunity**
14	Other	50	Prequalified by WHO§
15	Significant	50	Sustainability of the market availability of the vaccine and supplies in the longer term‡
**16**	**Significant**	**40**	**Outbreak potential incl. past occurrence of outbreaks and potential for international spread, and epidemic and pandemic risk**
17	Other	40	Loss of quality-adjusted life years‡
18	Other	40	Social and economic benefits, including reduction in healthcare costs, improvement in life expectancy, in quality of life for individuals, families, caregivers and communities, productivity gains¶
**19**	**Other**	**40**	**Impact on resistance to antibiotics and antivirals**
**20**	**Other**	**40**	**Effect of the vaccine on transmission**
**21**	**Other**	**40**	**Number needed to vaccinate to prevent a case**
22	Significant	30	Contribution to national/regional/global goals (eg, eradication, control, elimination, reduction)**
23	Significant	30	Direct and indirect costs to patients and families**
24	Other	30	Accessibility and equity of vaccination for the target population§
25	Essential	30	Availability of adequate cold chain equipment at all levels or the ability to procure cold chain equipment (CCE) required to store the vaccine§
**26**	**Essential**	**30**	**Acceptability of schedule (eg, multiple injections, additional visits)**
27	Significant	30	Burden inequity (highest prevalence in poorer/at-risk populations/gender inequity)**
28	Essential	30	Perception of the target population of the disease risk, severity, fear and demand for disease control**
**29**	**Other**	**30**	**Perspective on vaccine price**

Bold criteria were ultimately included as the final criteria.

* Essential, significant and other criteria were assigned weights of 3, 2 and 1, respectively.

† This criterion was merged with the first criterion.

‡ These criteria were excluded because they had an overlap with another higher-ranked criterion.

§ These criteria were excluded because they could not differentiate the selected vaccines.

¶ This criterion was excluded because it was considered too complex for adequate data collection.

** These importance criteria were tied with feasibility criteria and were excluded in favour of other feasibility criteria.

### Vaccine prioritisation

**Figure 2:**
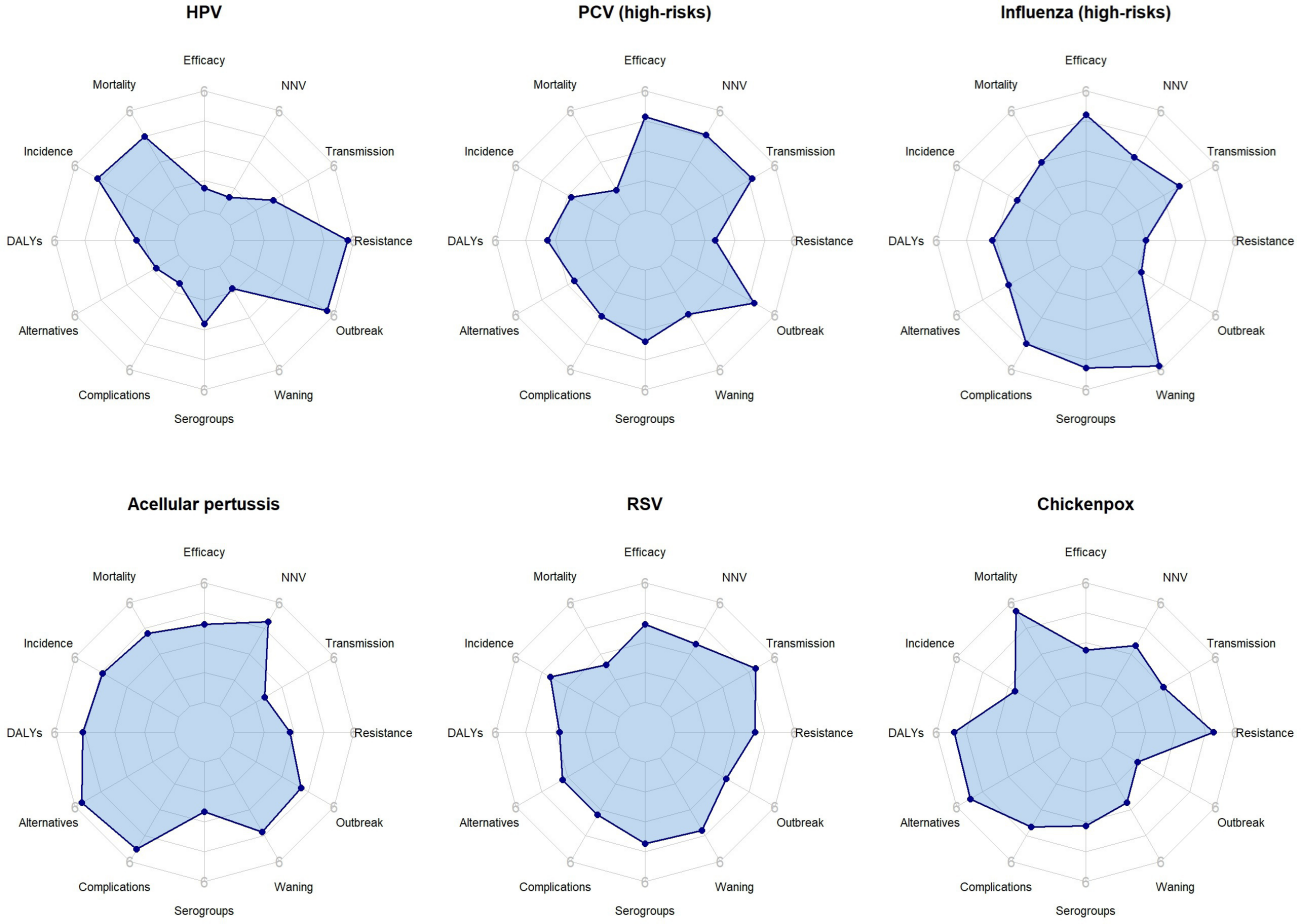
Radar plots of average vaccine rank for each importance criterion. For the complete names of the criteria, refer to table 2. The criteria, starting at 12 o’clock and proceeding anticlockwise, correspond to row ranks 1st, 2nd, 3rd, 5th, 6th, 7th, 12th, 13th, 16th, 19th, 20th and 21st. DALYs, disability-adjusted life years; HPV, human papillomavirus; NNV, number needed to vaccinate; PCV, pneumococcal conjugate vaccine; RSV, respiratory syncytial virus.

Average vaccine rankings for each criterion are presented in figures2  3, as well as in the online supplemental tables 2 and 3. Overall, HPV was ranked first in half of the importance and feasibility criteria.

**Figure 3:**
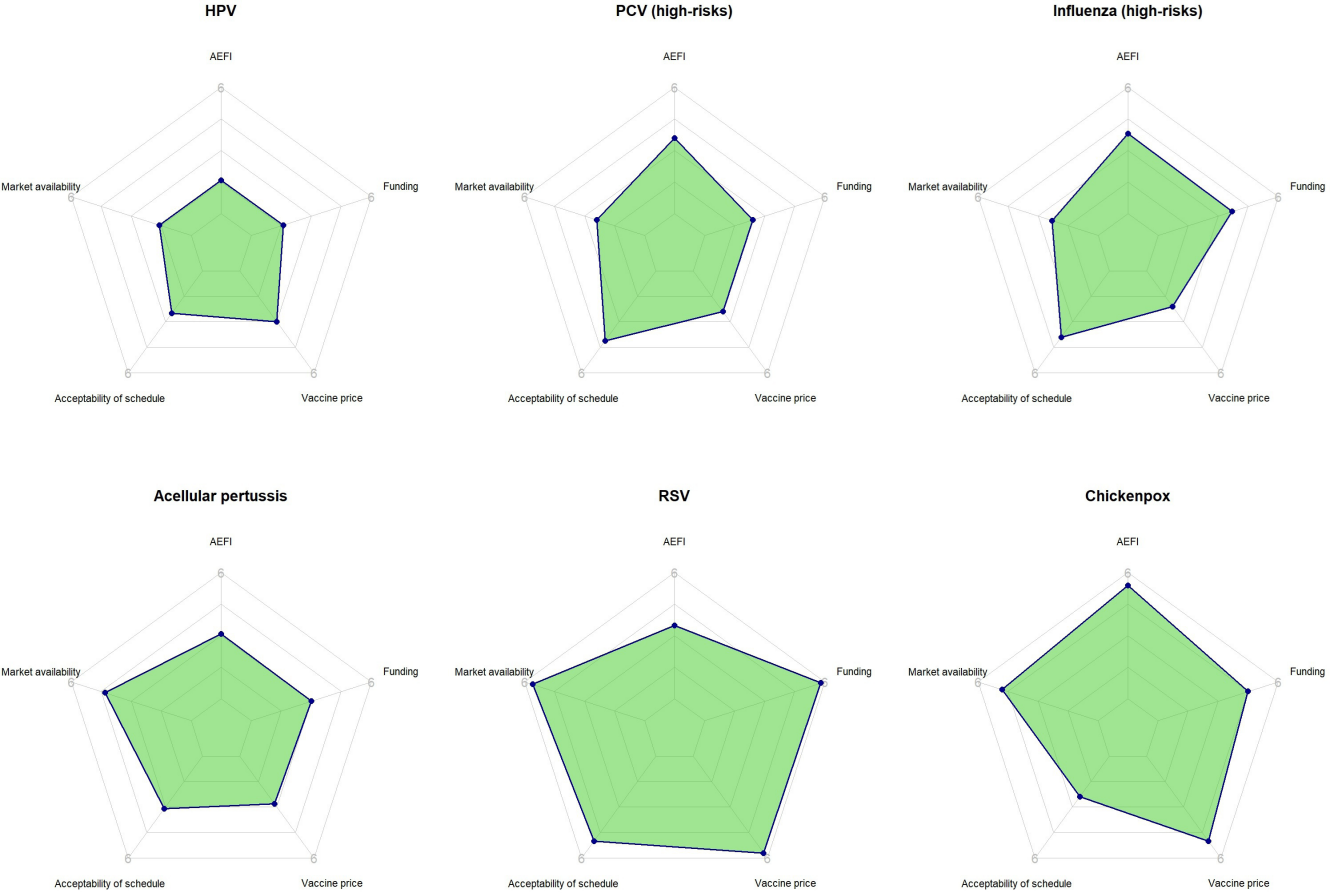
Radar plots of average vaccine rank for each feasibility criterion. For the complete names of the criteria, refer to table 2. The criteria, starting at 12 o’clock and proceeding anticlockwise, correspond to row ranks 4th, 8th, 26th, 29th and 9th. AEFI, adverse event following immunization; HPV, human papillomavirus; PCV, pneumococcal conjugate vaccine; RSV, respiratory syncytial virus.

Regarding vaccine benefits, HPV ranked highest overall, whereas influenza for high-risk groups ranked lowest. Specifically, HPV had average rankings of 1.1 for vaccine effectiveness and efficacy, 1.3 for vaccine waning of immunity and 2.7 for coverage of active serogroups. In contrast, influenza for high-risk groups had average rankings of 4.8–5.8 for the above criteria. Interestingly, HPV was ranked last (5.7) for the impact of vaccine on antimicrobial use, while influenza was ranked first (1.5). With respect to disease burden and epidemiology, HPV had average rankings of under 2.0 for disease complications and disability-adjusted life years, while also having average rankings of around 5.0 for disease mortality and incidence. PCV for high-risk groups and RSV had the highest rankings for mortality (1.4 and 2.4, respectively), while influenza for high-risk groups and chickenpox had the highest rankings for incidence (2.5 and 2.6, respectively) (online supplemental table 2). Moving to feasibility criteria, chickenpox and HPV vaccines demonstrated the most compatibility with the current vaccination schedule in Iran (2.4 and 2.5, respectively), while RSV and chickenpox vaccines demonstrated the worst rankings for vaccine price, market availability of the vaccine and the availability and sustainability of funding for the vaccine.

### Sequencing scenarios

**Figure 4:**
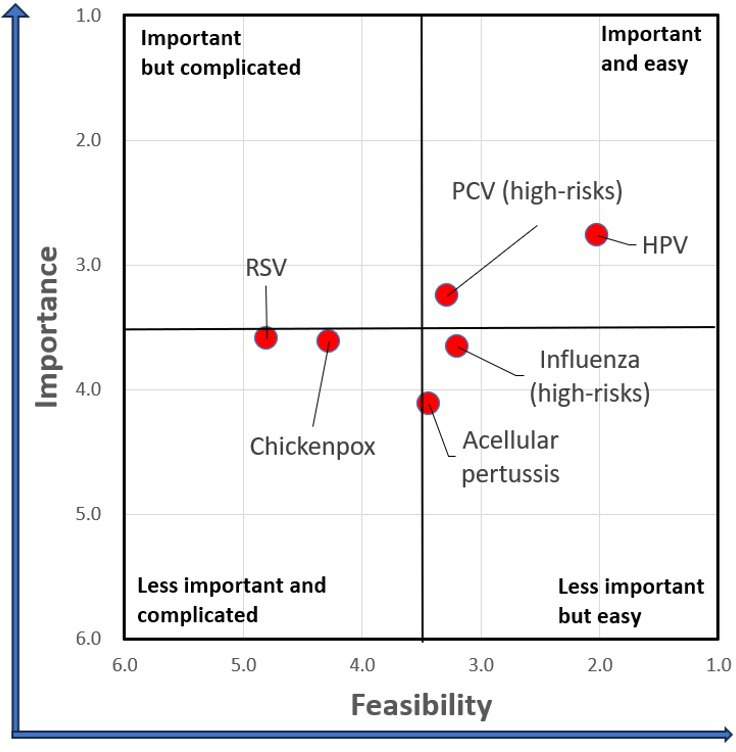
Four-quadrant matrix of average importance and feasibility rankings of the selected vaccines. HPV, human papillomavirus; PCV, pneumococcal conjugate vaccine; RSV, respiratory syncytial virus.

**Figure 5:**
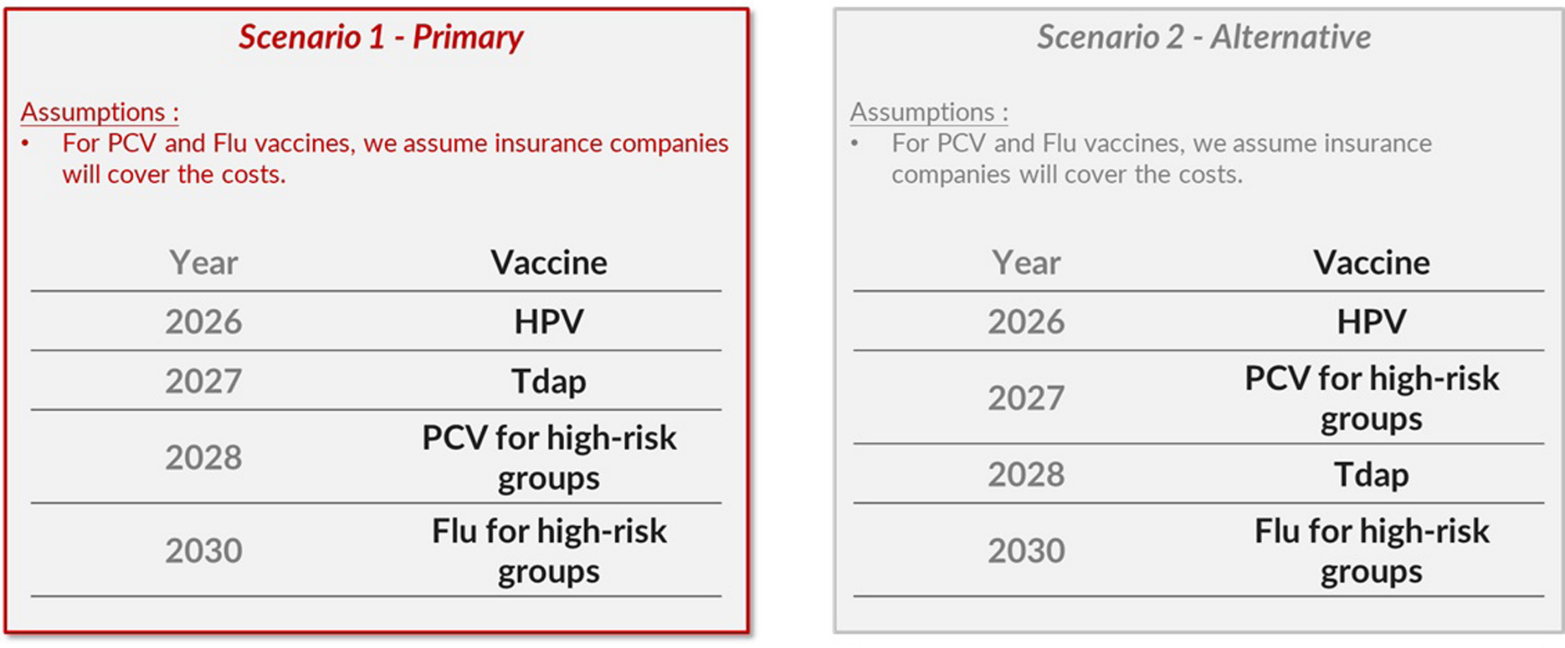
Recommended sequencing scenarios for the introduction of new vaccines in Iran, as proposed by the NITAG. HPV, human papillomavirus; NITAG, National Immunisation Technical Advisory Group; PCV, pneumococcal conjugate vaccine; Tdap, tetanus-diphtheria-acellular pertussis vaccine.

The weighted averages for importance and feasibility criteria are presented in table 3. RSV, chickenpox and influenza for high-risk groups had similar levels of importance but differed in their feasibility rankings. Conversely, PCV for high-risk groups, influenza for high-risk groups and aP shared comparable levels of implementation difficulty, with PCV for high-risk groups demonstrating a higher importance ranking. Overall, HPV was ranked first, followed by PCV and influenza for high-risk groups. On the second day of Workshop II, I-NITAG members used figure 4 as a basis for discussion on high-priority and medium-priority vaccines. There was a unanimous consensus on the placement of hexavalent and HPV vaccines as high-priority vaccines, and of RSV and chickenpox vaccines as low-priority vaccines. Following extended deliberations, I-NITAG proposed two prioritisation scenarios: in one, PCV for high-risk groups was designated as the fourth high-priority vaccine, whereas in the alternative, Tdap for high-risk groups occupied the fourth high-priority position (figure 5). The RSV and chickenpox vaccines were not recommended for introduction within the next 5-year horizon.

**Table 3:** Average weighted ranking of each vaccine across importance criteria, feasibility criteria and their combined ranks

Vaccine	Importance ranks	Feasibility ranks	Combined ranks
HPV	2.8	2.0	2.5
PCV (high-risks)	3.2	3.3	3.2
Influenza (high-risks)	3.7	3.2	3.5
Chickenpox	3.6	4.3	3.8
Acellular pertussis	4.1	3.4	3.9
RSV	3.6	4.8	4.0

HPV, human papillomavirus; PCV, pneumococcal conjugate vaccine; RSV, respiratory syncytial virus.

## Discussion

This study applied WHO’s NVI-PST to help Iran’s NITAG establish a transparent, evidence-based roadmap for introducing new vaccines over 2025–2030. The process was nationally led, adapted to context and implemented through two workshops that finalised 17 criteria, compiled comprehensive evidence dossiers, scored vaccine candidates on importance and feasibility, and then developed priority tiers and sequencing scenarios. The outputs were endorsed in principle by MoHME and disseminated to policymakers and professional audiences.

From a policy perspective, this is among the first documented applications of the NVI-PST in the WHO Eastern Mediterranean Region, filling a gap in the published literature. As such, the work provides a replicable example of structured, evidence-to-decision processes for multivaccine prioritisation in the Region and strengthens Iran’s capacity for life-course immunisation planning.

Findings from this exercise align with multicriteria prioritisation work in Bangladesh, China, the Republic of Korea and Thailand, yet each used a distinct engine. Bangladesh applied a workshop-based MCDA: criteria were shortlisted and ranked, weights derived from rank-order centroid, then refined by direct rating and a few context-sensitive dimensions were left for deliberation rather than strict scoring.^5^ China also used MCDA, but with a formal value-measurement approach (MACBETH, Measuring Attractiveness by a Categorical Based Evaluation Technique), converting pairwise qualitative judgements into cardinal weights and value functions, and testing robustness through sensitivity analyses—powerful but technically demanding.^6^ Korea prioritised governance and evidence readiness: candidates were screened for national data availability, then ranked through a modified Delphi process with anonymous, multiround expert surveys, emphasising consensus under explicit minimum-evidence standards.^7^ Thailand elicited criterion importance using best-worst scaling, yielding a clean weight structure across stakeholder groups with low cognitive burden, then applied those weights in subsequent ranking.^8^

Against this backdrop, our work applied the WHO NVI-PST in Iran, combining structured criteria with a dual-pillar design and an explicit sequencing objective. By dual-pillar, we mean scoring each vaccine on two coequal dimensions, importance (public-health impact) and feasibility (programme practicality), and using their joint position, rather than a single composite score, to guide tiering and multiyear sequencing. In contrast to Bangladesh and China’s single-index MCDA, Korea’s Delphi-centric consensus and Thailand’s preference-elicitation focus, the WHO NVI-PST, as implemented in Iran, kept both pillars visible, applied live, transparent scoring to full evidence dossiers and translated results directly into executable timelines.

### Advantages of the framework used

Beyond yielding a prioritised list, the NVI-PST provided a disciplined way to practise evidence-informed decision-making. The toolkit insisted on a limited, agreed set of criteria and indicators, explicit decision rules, documented voting and an audit trail of evidence and rationale—all of which increase transparency and reproducibility for future cycles.

The process also helped balance influence and ensure more even participation. By using structured, criterion-by-criterion ranking with live polling and predefined decision methods, the group could temper the effect of highly vocal members and anchor deliberation in comparable evidence displays. The read-me explicitly recommends online tools with traceable ballots and automatic aggregation, which Iran operationalised; voting and analysis produced importance-feasibility quadrants that made trade-offs visible and sped consensus.

Clarity of process was another asset. The toolkit’s slide templates, ranking model and evidence-collection guides standardised how data were gathered and presented, enabling participants to compare vaccines consistently across criteria and days. In Iran, the team compiled full evidence dossiers for each vaccine, covering the 17 agreed criteria, which underpinned the credibility of the final tiers and scenarios.

Practical job aids within NVI-PST, especially the slide decks, calculation workbook and instructions for live polling, materially smoothed execution. The toolkit emphasises preparing ‘evidence overview’ slides for each criterion and integrating links/QR codes to the ranking tool; those features kept the flow efficient and participant engagement high.

Finally, online voting and ranking helped streamline the workshops, reduced bias and deepened participation by turning subjective impressions into traceable, aggregated ranks that could be discussed transparently.

### Limitations of the tool and process

Even with these strengths, the full cycle was time-intensive and labour-intensive. Conducting a national adaptation workshop, building complete evidence dossiers across 17 criteria for multiple vaccines, running a 2-day scoring workshop and then crafting sequenced recommendations required sustained effort from a core team and extensive stakeholder time. The final report explicitly documents the breadth of activity and deliverables required across over 6 months, a heavy lift that many countries will only manage with strong coordination.

A functioning secretariat or project team was indispensable. In Iran, the implementation team coordinated surveys, prepared materials, compiled and quality-checked evidence, conducted calculations, facilitated workshops and packaged the recommendations, roles that typical volunteer NITAG members could not realistically take on in addition to their clinical and academic duties.

### Modifications we made in practice

Iran’s NITAG ultimately used 17 criteria, exceeding the recommended 10–12 targets, because members sought adequate representation of both the ‘importance’ and ‘feasibility’ dimensions. While this expanded set better reflected the committee’s priorities, it increased the evidence-collection burden and extended discussion time in Workshop 2 as more criteria required presentation and ranking.

Maintaining a minimum representation of feasibility criteria proved challenging. Stakeholders tended to emphasise importance criteria in their rankings, so the team had to ensure sufficient feasibility criteria were included and weighted to prevent a purely importance-oriented ordering that would ignore the feasibility of introducing new vaccines, which is more important to the MoHME authorities. This mirrors experiences in other countries where disease burden and vaccine effectiveness often dominate without deliberate balance.

Given members’ limited time and competing obligations, we compressed the first workshop to a single day with a follow-up virtual session and ran the second workshop over 2 days instead of 3. To make that feasible, we invested in meticulous prework: standardised slide packs, pretested polling and precalculated aggregation models so that live sessions focused on interpretation and consensus, not mechanics.

A dedicated project team took on time-consuming tasks that could not be distributed to NITAG members, including literature review and synthesis, running the virtual voting stack and performing rank analysis. This division of labour was decisive for on-time delivery and data quality.

### Recommendations to strengthen the tool and process

Expanding and maintaining the WHO Vaccine Evidence Compendium to assist NITAGs would substantially reduce the country’s workload. The most time-consuming step was assembling up-to-date, indicator-ready evidence across multiple vaccines; a broader compendium would shorten timelines and improve comparability across settings.

It may be more practical to frame criteria into two layers, importance and feasibility, and require a minimum from each, rather than classifying criteria into ‘essential/significance/other’ with subjective weights. In our experience, the latter added complexity while having limited effect on the final ordering compared with the clear signal that came from the side-by-side ranking within the importance/feasibility matrix.

The key take-away messages from Iran’s experience are that the NVI-PST proved fully feasible under genuine national leadership, delivering credible and actionable roadmaps even with a largely volunteer-based NITAG; that sustainable adoption in low- and middle-income settings will require streamlining evidence sources, simplifying weighting rules and strengthening secretariat support; and that structured multi-vaccine prioritisation offers broader value for aligning immunisation policies, sharing experiences and fostering collaboration across the Eastern Mediterranean Region.

These targeted refinements would transform the NVI-PST into a lighter, routinely repeatable tool for evidence-informed immunisation planning.

## Conclusion

This nationally led application of the NVI-PST produced a credible, consensus-based prioritisation that elevates HPV and PCV for high-risk groups into the high-priority tier, with practical sequencing scenarios for the medium-term horizon. As one of the earliest documented implementations of the toolkit in the Eastern Mediterranean Region, it demonstrates that countries can operationalise a repeatable framework that is both rigorous and adaptive to context. With modest refinements, especially streamlining evidence access and emphasising balanced importance-feasibility coverage, the tool can provide a stable platform for periodic reassessment as epidemiology, markets and budgets evolve.

## Supplementary material

10.1136/bmjopen-2025-115580 online supplemental file 1

## Data Availability

Data are available upon reasonable request.
