# Supplementary material for: Application of the WHO new vaccine introduction prioritisation and sequencing framework to guide evidence-based vaccine introduction decisions in Iran, 2025–2030
Source: BMJ Open. 2026 Apr 15;16(4):e115580. doi: 10.1136/bmjopen-2025-115580 (PMC13084812; doi:10.1136/bmjopen-2025-115580)
Supplement: online supplemental file 1 [file bmjopen-16-4-s001.docx]

**Supplementary Table 1** Evaluated indicators for the selected criteria

| **Criteria** | **Indicators** |
| --- | --- |
| Effectiveness of the vaccine including in different populations/age groups/cohorts AND^1^ Efficacy and immunogenicity of the vaccine in target population | Efficacy against Infection, Hospitalization, Severe Disease and Death |
|  | Effectiveness against Infection, Hospitalization, Severe Disease and Death |
| Mortality and lethality including in different sociodemographic and age groups | Case Fatality Rate |
|  | Infection Fatality Rate |
|  | Mortality rate |
| Incidence including in different sociodemographic and age groups | Incidence including in different sociodemographic and age groups |
| Risk at individual level incl. Type, severity, consequences and frequency of AEFI, including reactogenicity profile & capacity to mitigate known adverse events | Frequency by Type (e.g., local, systemic, allergic, neurological, anaphylaxis, etc.) |
|  | Frequency by Severity (mild, moderate, or severe using standard definitions like WHO or Brighton Collaboration) |
|  | Frequency by Consequences (e.g., recovery without sequelae, hospitalization, disability, death) |
| Disability-adjusted life years (DALYs) | DALYs in different sociodemographic and age groups |
| Absence of satisfactory alternatives to prevent/treat the disease (considering effectiveness, cost and practicality) | Effectiveness of existing alternatives |
| Long-term complications of disease (e.g. frequency of survivors with sequelae) | Frequency of Long-term Complications (No complication, Cancer, Alzheimer’s, etc.) (reversible or irreversible) |
| Market availability of the vaccine and supplies over the selected time period | Global demand |
|  | Number of suppliers |
|  | Past reports of stockout |
|  | Global supply |
| Availability and sustainability of funding to cover the total cost of the program (incl. GAVI eligibility) | Evaluation of national budget for stability and predictability over the long term |
| Coverage of active serogroups or serotypes in the country (for serogroup- or serotype-specific vaccines) | Does it have different serogroups/serotypes? |
|  | Vaccine coverage of Circulating or Significant Serogroups/Serotypes by the Vaccine |
| Duration of protection and waning of immunity | Booster Requirement Status |
|  | Vaccine effectiveness and Seropositivity rate over time |
| Outbreak potential incl. past occurrence of outbreaks and potential for international spread, and epidemic and pandemic risk | History of Outbreaks in the Country: Number, size, and frequency of past outbreaks of the disease in the country |
| Impact on resistance to antibiotics & antivirals | Impact on resistance to antibiotics & antivirals |
| Effect of the vaccine on transmission | Secondary attack rate (SAR) reduction (vaccine effectiveness against transmission) |
| Number needed to vaccinate to prevent a case | NNV to Prevent One Case (Infection, Hospitalization, Severe Disease, Death) |
| Acceptability of schedule (e.g. multiple injections, additional visits) | Total number of doses/visits required |
|  | Number of injections per visit |
|  | Interval between doses |
|  | Synchronization with existing immunization visits (can it be co-administered with existing vaccines?) |
| Perspective on vaccine price | Price per course |

**Supplementary Table 2** Average vaccine rankings across importance criteria

| **Vaccine/Criterion** | **Efficacy** | **Mortality** | **Incidence** | **DALYs** | **Alternatives** | **Complications** | **Serogroups** | **Waning** | **Outbreak** | **Resistance** | **Transmission** | **NNV** |
| --- | --- | --- | --- | --- | --- | --- | --- | --- | --- | --- | --- | --- |
| HPV | 1.1 | 4.5 | 4.7 | 1.9 | 1.3 | 1.0 | 2.7 | 1.3 | 5.6 | 5.7 | 2.5 | 1.0 |
| Influenza  (high-risks) | 4.8 | 3.0 | 2.5 | 3.2 | 3.0 | 4.5 | 4.9 | 5.8 | 1.7 | 1.5 | 3.9 | 3.3 |
| PCV  (high-risks) | 4.7 | 1.4 | 2.8 | 3.4 | 2.6 | 2.9 | 3.6 | 2.8 | 4.8 | 2.0 | 4.7 | 4.6 |
| Acellular  pertussis | 3.9 | 4.2 | 4.4 | 4.6 | 5.6 | 5.3 | 2.5 | 4.3 | 4.1 | 2.8 | 2.0 | 4.9 |
| RSV | 3.9 | 2.4 | 4.0 | 2.8 | 3.3 | 3.3 | 4.1 | 4.2 | 3.2 | 4.0 | 4.9 | 3.6 |
| Chickenpox | 2.6 | 5.5 | 2.6 | 5.1 | 5.2 | 4.0 | 3.2 | 2.6 | 1.5 | 4.9 | 3.0 | 3.5 |

**Supplementary Table 3** Average vaccine rankings across feasibility criteria

| **Vaccine/Criterion** | **AEFI** | **Market availability** | **Acceptability of schedule** | **Vaccine price** | **Funding** |
| --- | --- | --- | --- | --- | --- |
| HPV | 1.6 | 1.6 | 2.5 | 3.0 | 1.6 |
| Influenza  (high-risks) | 3.8 | 2.3 | 3.9 | 2.1 | 3.7 |
| PCV  (high-risks) | 3.6 | 2.4 | 4.1 | 2.4 | 2.4 |
| Acellular  pertussis | 3.1 | 4.3 | 3.1 | 2.8 | 3.0 |
| RSV | 3.5 | 5.6 | 5.0 | 5.7 | 5.8 |
| Chickenpox | 5.4 | 4.8 | 2.4 | 5.0 | 4.5 |
