# Supplementary material for: Application of the WHO new vaccine introduction prioritisation and sequencing framework to guide evidence-based vaccine introduction decisions in Iran, 2025–2030
Source: BMJ Open. 2026 Apr 15;16(4):e115580. doi: 10.1136/bmjopen-2025-115580 (PMC13084812; doi:10.1136/bmjopen-2025-115580)
Supplement: Supplementary file 2 [file bmjopen-2025-115580.reviewer_comments.pdf]

PEER REVIEW HISTORY

BMJ Open publishes all reviews undertaken for accepted manuscripts. Reviewers are asked to complete a checklist review form and are provided with free text boxes to elaborate on their assessment. These free text comments are reproduced below.

ARTICLE DETAILS

Title (Provisional)

Application of the WHO New Vaccine Introduction Prioritization and Sequencing Framework to Guide Evidence-Based Vaccine Introduction Decisions in Iran, 2025 - 2030

Authors

Fotouhi, Akbar; Younesian, Sobhan; Mousavi, Seyede Maryam; Zahraei , Seyed Mohsen; Mahmoudi, Sussan; Fotouhi, Farid; Nojomi, Marzieh; Alavi, Maryam; Zamani, Omid; Sedighi, Iraj; Nateghian, Alireza; AkbariSari, Ali; Movahedi, Masoud; Gharagozlou, Mohamad; Mamishi, Setareh; Salehi, Mohammadreza; Biglari, Alireza; Es-Haghi, Ali; Moradi, Ghobad; Gouya, Mohammad-Mehdi

---

VERSION 1 - REVIEW

**Reviewer**	**1**
**Name**	**Amaral, Gabriela**
**Affiliation**	**Universidade de Sao Paulo, Enfermagem em Saúde Coletiva**
**Date**	**08-Feb-2026**
**COI**	**None**

Dears authors,

I hope this message finds you well.

I would like to congratulate you on the manuscript, which addresses a topic of relevance to public health in Iran and provides important guidance for the introduction of new vaccines into the National Immunization Program. I have identified several points that require revision to enhance clarity, objectivity, and the scientific consistency of the work. The considerations presented aim to guide the refinement of the text, strengthen the arguments, and ensure greater methodological and conceptual precision, thereby contributing to the manuscript's quality and increasing its relevance and scientific contribution.

Sincerely,

1. GENERAL GUIDANCE:

• There is extensive use of acronyms throughout the manuscript. Some, although previously defined, are spelled out again in later sections, while others are mentioned only once (e.g., CDC) and could be retained only in full form. I recommend reviewing the use of acronyms to standardize their presentation and eliminate redundancies.

2. ABSTRACT:

• Revision and adjustment are necessary to ensure the abstract faithfully reflects the modifications suggested throughout the manuscript.

3. INTRODUCTION:

• The introduction is well-structured and clearly written. It provides appropriate background, clearly defines the research problem, and presents the study objective and rationale in a coherent manner, supporting the relevance of the investigation.

4. METHODS:

• The methods are well described and demonstrate adequate methodological rigor. However, minor adjustments and clarifications could enhance transparency and reproducibility:

o (2.1) Study design and governance: Although the manuscript includes a section titled "Study design," the study type is not explicitly defined. I recommend clearly indicating the study design to improve methodological transparency and facilitate reader comprehension.

o (2.3) Phase 1: Framework adaptation: The main decision-makers and stakeholders included in the panel are presented. However, the number of participating members is not reported. I recommend specifying the number of participants involved in this phase to enhance methodological transparency.

5. RESULTS:

• The results are clearly presented and consistent with the proposed study methodology, allowing an adequate understanding of the findings in light of the stated objectives.

6. DISCUSSION:

• The authors provide a comprehensive overview of the results and discuss them appropriately in light of studies conducted in other settings, such as Bangladesh, China, the Republic of Korea, and Thailand, contributing to contextualization of the findings and dialogue with the existing literature.

**Reviewer**	**2**
**Name**	**Dadari, Ibrahim**
**Affiliation**	**UNICEF**

**Date**	**11-Feb-2026**
**COI**	**None**

This manuscript presents a timely and policy-relevant documentation of Iran's nationally led application of the WHO New Vaccine Introduction Prioritization and Sequencing Toolkit (NVI-PST) to guide new vaccine introduction prioritization for 2025–2030. The paper demonstrates a transparent description of a structured, criteria-based prioritization process; explicit separation of importance and feasibility dimensions; use of predefined weighting and anonymous voting to enhance deliberative rigor; and clear articulation of sequencing scenarios aligned with programmatic constraints. The manuscript contributes novel regional evidence from the WHO Eastern Mediterranean Region (EMRO) and offers practical insights for NITAGs seeking systematic multi-vaccine prioritization approaches through the Vaccine Prioritization and Optimization exercise (VPOp). The methods are generally well described, though clarification on the handling of ordinal ranking, weighting rationale, and robustness checks could strengthen methodological transparency. The discussion appropriately reflects implementation challenges, particularly evidence gaps and secretariat workload, although further elaboration on policy implications (e.g., implications for life-course immunization planning and financial sustainability) would enhance impact. Overall, this is a well-structured, credible, and policy-relevant contribution that will, in no small measure, contribute to the regional and global prioritization discourse.

VERSION 1 - AUTHOR RESPONSE

Reviewer 1

We thank Reviewer 1 for the careful reading and constructive suggestions.

1. GENERAL GUIDANCE

Comment: There is extensive use of acronyms throughout the manuscript. Some, although previously defined, are spelled out again in later sections, while others are mentioned only once (e.g., CDC) and could be retained only in full form. I recommend reviewing the use of acronyms to standardize their presentation and eliminate redundancies.

Response:
We have comprehensively reviewed the manuscript for consistency in acronyms. Specifically, we removed redundant redefinitions of previously defined acronyms, spelled out acronyms that were used only once (e.g., CDC), ensured uniform use of NVI-PST, I-NITAG, EPI, MoHME, etc.

2. ABSTRACT

Comment: Revision and adjustment are necessary to ensure the abstract faithfully reflects the modifications suggested throughout the manuscript.

Response:
The abstract has been revised in accordance with the modifications.

3. INTRODUCTION

Comment: The introduction is well-structured and clearly written. It provides appropriate background, clearly defines the research problem, and presents the study objective and rationale in a coherent manner, supporting the relevance of the investigation.

Response:
We thank the reviewer for the encouraging feedback.

4. METHODS

4a. Comment: (2.1) Study design and governance: Although the manuscript includes a section titled "Study design," the study type is not explicitly defined. I recommend clearly indicating the study design to improve methodological transparency and facilitate reader comprehension.

Response:
We have now explicitly defined the study design in Section 2.1 as:

"This study is a policy implementation case study applying a structured multi-criteria decision analysis (MCDA)-informed prioritization framework within a national immunization governance process."

4b. Comment: (2.3) Phase 1: Framework adaptation: The main decision-makers and stakeholders included in the panel are presented. However, the number of participating members is not reported. I recommend specifying the number of participants involved in this phase to enhance methodological transparency.

Response:
We thank the reviewer for this important suggestion. We have revised the Methods section to explicitly report the number and composition of participants in each meeting phase.

Specifically:

- The initial virtual session (25 February 2025) included 16 participants, comprising 11 core I-NITAG members, 5 non-core members, and stakeholders.
- Workshop I (21 May 2025) included 21 participants, comprising 10 core I-NITAG members, 11 non-core members, and stakeholders.
- Workshop II (19–20 August 2025) included 19 participants, comprising 11 core I-NITAG members, 8 non-core members, and stakeholders.

These details have been added to Sections 2.3 and 2.4.2 of the revised manuscript.

5. RESULTS

Comment: The results are clearly presented and consistent with the proposed study methodology, allowing an adequate understanding of the findings in light of the stated objectives.

Response:
We thank the reviewer.

6. DISCUSSION

Comment: The authors provide a comprehensive overview of the results and discuss them appropriately in light of studies conducted in other settings, such as Bangladesh, China, the Republic of Korea, and Thailand, contributing to contextualization of the findings and dialogue with the existing literature.

Response:
We appreciate the reviewer's acknowledgment.

Reviewer 2

Comments to the Author:
This manuscript presents a timely and policy-relevant documentation of Iran's nationally led application of the WHO New Vaccine Introduction Prioritization and Sequencing Toolkit (NVI-PST) to guide new vaccine introduction prioritization for 2025–2030. The paper demonstrates a transparent description of a structured, criteria-based prioritization process; explicit separation of importance and feasibility dimensions; use of predefined weighting and anonymous voting to enhance deliberative rigor; and clear articulation of sequencing scenarios aligned with programmatic constraints. The manuscript contributes novel regional evidence from the WHO Eastern Mediterranean Region (EMRO) and offers practical insights for NITAGs seeking systematic multi-vaccine prioritization approaches through the Vaccine Prioritization and Optimization exercise (VPOp). **The methods are generally well described, though clarification on the handling of ordinal ranking, weighting rationale, and robustness checks could strengthen methodological transparency.** The discussion appropriately reflects implementation challenges, particularly evidence gaps and secretariat workload, although further elaboration on policy implications (e.g., implications for life-course immunization planning and financial sustainability) would enhance impact. Overall, this is a well-structured, credible, and policy-relevant contribution that will, in no small measure, contribute to the regional and global prioritization discourse.

Response:
We thank the reviewer for this constructive suggestion. To strengthen methodological transparency, we have expanded Section 2.4.2 of the Methods to clarify three aspects of the aggregation process:

1. **Handling of ordinal rankings:** We now explicitly state that participants assigned ordinal ranks (1 = highest priority), that rankings reflect relative positioning rather than equal-interval differences, and that mean rank aggregation was used as a structured group summarization approach consistent with multi-criteria deliberative processes.
2. **Weighting rationale:** We clarified that weights (3, 2, 1) were derived from the NVI-PST classification of criteria into essential, significant, and other categories, which were determined during Phase 1 based on importance, data availability, and discriminative capacity.
3. **Robustness assessment:** We added a description of how ranking stability was reviewed by comparing weighted and unweighted results and by examining quadrant clustering during deliberation. Although no formal quantitative sensitivity analysis was conducted, we clarify that final prioritization decisions were not based solely on marginal numerical differences.

VERSION 2 - REVIEW

**Reviewer**	**1**
**Name**	**Amaral, Gabriela**
**Affiliation**	**Universidade de Sao Paulo, Enfermagem em Saúde Coletiva**
**Date**	**07-Mar-2026**
**COI**	

Dear Authors,

I hope this message finds you well.

The revisions made by the authors in response to my previous comments have been carefully reviewed. After verifying the modifications implemented following the revision round, I would like to congratulate the authors for the care taken and the quality of the work presented. The revisions satisfactorily addressed the recommendations provided, and therefore I recommend the acceptance of the manuscript for publication.

Sincerely,
